# Surgical Cases

**Published:** 1858-11

**Authors:** A. Lopez

**Affiliations:** Mobile, Alabama


### ART. II.—*Surgical Cases.* By A. LOPEZ, M.D., of Mobile, Alabama.

CASE I.—*Injury of the Forearm, with Extensive Destruction of Parts; Reçovery without Amputation.*—Gabriel, a black lad, aged sixteen, was an attendant in a rice-mill on a plantation, where his duty was to feed the mortars. In order to explain the character of the injury, I will describe a portion of the apparatus concerned. There are two uprights, across which, about midway, there is a large horizontal beam or girder. Through this latter, at various distances, are mortices for the transit of the pestles, which fit as perfectly in them as possible, allowing only for the free passage up and down, to and from the mortar beneath. The mill being at full speed, Gabriel was at his station, feeding the mortars, and, as he was exhausted, leaned his head and left arm against this middle beam or girder, in which position he nodded, while mechanically discharging his duty. He ultimately fell asleep, and slipped from his position just as the pestle was ascending. The act instinctively caused him to throw forward his right hand. As he fell, the palm of his hand came in contact with the inferior ascending portion of the pestle, and before he recovered himself, the whole hand and arm were carried up through the mortice, between its inner surface and the pestle. The most extraordinary circumstance is, that the space through which his arm passed could not, while the mill was at rest, receive more than the blade of a case-knife introduced flatwise.* The wheel was stopped as quickly

* See *Amer. Jour. Med. Sci.*, No. xlii., p. 387, February, 1837, for an account of "Arm and Shoulder Torn from the Body," reported by Dr. Mussey, who, after detailing the accident and lesion, says,—"I measured accurately the space between as possible, and it was then found impracticable to extricate him except by sawing through the timber on each side of his arm, which was tightly wedged. As soon as the removal was accomplished, the usual domestic remedies were applied, and the limb tightly wrapped in several bandages, when a message summoned me to the plantation, distant between three and four miles. On my arrival, he was lying beneath the shade of a tree, with as little suffering as usually attends such vast destruction and its shock. He was placed on a mattress in a cart, and carried to town slowly and carefully, giving him, previous to his departure, one hundred drops of laudanum, as I feared the effects of the injury upon the negro idiosyncrasy. The envelopes being removed, the following condition was exhibited. The palmar surface of the wrist was cut deeply through the annular ligament; the wound extending from the radial to the ulnar side. The radial artery at the pulse was unhurt. On the upper and outer portion of the forearm there was a total destruction of the fleshy and tendinous structures from their insertions and interosseal attachments upward, to their origin at the two condyles of the humerus. The parts involved were—1. The extensor carpi radialis; 2. Extensor carpi radialis brevior; 3. Extensor pollicis; 4. Extensor digitorum communis; 5. Extensor carpi ulnaris. These muscles, with their entire tendinous attachments, were stripped from their osseous and other connections as though they had been scraped with a planing machine. All the minor arteries and veins with their ramifications were destroyed, and the radial and ulnar nerves with their twigs torn to shreds. The radius was fractured transversely at two portions of its shaft, distant from each other about four inches. The tattered condition of the limb presented a most unfavorable prospect for successful experiment, yet we were compelled to try, as permission was peremptorily refused to have his limb removed. We as urgently recommended it, pointing to the extent of the lesion, the thorough destruction of parts, and excessive heat of the weather. We told him of the liability to lockjaw, and to the whole train of constitutional symptoms, but beyond all, our own responsibility. Notwithstanding, he insisted on an attempt to save the limb, and we yielded, "*par force.*" It is needless to detail the various means pursued from stage to stage; nay, frequently from hour to hour, driven as we were by an intensely high thermometrical range, to combat constitutional irritation and mortification. We ran through the list of antiseptics externally as well as internally applied. The most important and efficient agents were yeast and

---

the drum or cylinder (of the mill in a cotton factory) and the ceiling, *through which he passed several times*, as before stated, and found it to be *scarcely seven inches and a half.*"

nitric acid, but I ascribe the successful issue chiefly to the free use of pyroligneous acid, one part to three of water.

By the most undeviating personal attention, three times daily from the date of the accident to the 24th of September ensuing, we finally triumphed in carrying the patient through the successive processes of sloughing, moderating suppuration, (which once or twice threatened seriously from its profusion,) and promoting granulation, and at last cicatrization. Once during the treatment, in the early stage, owing to high reaction, we were compelled to resort to venesection and antiphlogistics.

The fractured portions of bone were thrown off. When we dismissed Gabriel, we certainly were well pleased with the result, as he had an arm which, if not perfect in form or usefulness, was certainly more sightly than a stump, and it moreover added another to many proofs that there are numerous cases where the surgeon's knife, like the patriot's sword, should never be unsheathed until all constitutional means have failed.

CASE II.—*Injury of the Eye: Rupture of the Sclerotica by a Blow; Separation of the Iris from its Ciliary Connections; Extensive Extravasation; Adventitious Pupil.*—Kelly, an Irish laborer, during a drinking frolic, engaged in a fight, was knocked down, and received while on the ground a kick in the ball of the left eye, from the pointed shoe of his antagonist. This occurred on the 13th of March, 1841. He remained at his lodgings without aid until the 15th, when he sought my advice.

The inferior and superior palpebræ externally were entirely and deeply ecchymosed; and the cornea, sclerotica, and conjunctiva one chaotic mass, totally undistinguishable as an eye. It presented the appearance of a lump of currant jelly held before a strong light. The conjunctival linings of the upper and lower lids were heavily laden with extravasated blood, partly coagulated and partly fluid, hanging down over the ball in bags. Acute lancinating pains shot through the orbit. The brightest rays of light from either the sun or a candle were inappreciable.

I evacuated the extravasated blood by extensive scarifications, removed the coagula, and ordered continued applications of pledgets wet with a solution of acetate of lead during the day.

16th.—Ordered six leeches to the eyelids and temple, and compound cathartic powders, with strict diet, gruel and iced water.

17th.—Leeches drew well; powder operated freely; less pain and tension; no vision. Wet cups to the temporal regions; repeat purge; continue lotion.

18th.—Obtained by cupping yesterday five ounces of blood; powder operated; no vision; slight absorption. Ordered four grains of bichloride of mercury to four ounces of distilled water to be dropped tepid into the eye three times a day. I scarified again, and encouraged the depletion by warm bathing.

19th.—Absorption still progressing, but very slowly. By darkening the room and placing a lighted candle just before the eye, he is slightly sensible of its impression. Ordered blister for the neck; scarify again; continue eye-wash.

20th.—Blister drawn and discharges freely; absorption still going on; can indistinctly distinguish the shadow of my hand moved to and fro, although he cannot tell what the object is. Continue eye-water.

21st, 22d, 23d, 24th.—Same as on 20th. I scarified the eye on the 23d, and increased the wash by adding two grains of the mercury to the ounce of water.

25th.—Eye much less injected; absorption proceeding. Being a cloudy day, and his bowels not having been moved for three days, he does not see as well to-day. Ordered saline aperient; change wash for four grains of nitrate of silver to the ounce of water. I applied by means of a hair-pencil tinct. iodine (℈ij to ℥i alcohol) over the entire surface of the external orbit and around the temporal bone. In less than three minutes it burnt intensely, and continued so for a half hour.

26th.—Says he saw better last night than at any time since the injury. He could distinguish the walls and furniture of his chamber. Continue collyrium; repeat tinct. iodine, diluted one-third.

27th.—Materially improved; distinguishes a gold pencil-case. I held up a stethoscope, which he said looked like a flute, which proved that he saw it. Continue collyrium; insert a seton in his neck. To-day the absorption permitting a clearer inspection of the parts, I placed him in a strong light, and by means of a powerful lens, I perceive that the substance of the iris is to a greater or less extent absorbed; the pupil has undergone a deviation from its normal situation at the centre, being thrown upward upon the circumference of the membrane, assuming a semilunar or crescentic form; it is movable.

28th.—Better; distinguishes the books in my library, also a pen held up to him at the distance of two feet. Before he can decide upon the object held up for inspection, he has to use some varied movements of the head, so as to fix the axis of vision. The seton discharges. Continue collyrium; apply tinct. iodine, diluted one-half; saline aperient solution.

29th.—Salts operated; seton not discharging freely; smear it with ungt. basilicon, and apply warm poultice over it. He distinguishes to-day a small pill-box eighteen inches from the eye, also the male and female figures of an anatomical plate. The iodine yesterday burnt less; ecchymosis nearly disappeared; the separation of the iris from its ciliary connections is now perceptible. Repeat collyrium.

30th.—Increase collyrium; two grains of the salt to the ounce of water.

31st.—Can read the name on my sign. Continue collyrium.

April 1st.—Seton discharges profusely. Continue wash. The seton being nearly exhausted and harsh, insert a fresh skein of silk.

6th.—Seton discharging; eye free from injection; lids have resumed their natural size and appearance; eye lachrymose. Suspend collyrium and substitute sulphate of zinc, four grains to the ounce of water, to be used three times a day. He continued under treatment a week longer, and was dismissed cured. He sees as well as ever, and has escaped the amaurosis so common in such injuries, for doubtless the retina was severely concussed.

In all of my trials to prove his sight, I was careful to guard against deception by securing the sound eye. I attribute material benefit to the tinct. iodine, from the fact that the absorption of fluids extravasated in the chambers of the eye did not commence, nor proceed so decidedly, until it was employed.

CASE III.—*Physiological Surgery after Amputation.*—James, a negro, was shot, while in the woods, the contents of the gun entering his arm and forearm, destroying the bony and muscular structures of the extremity to within four inches of its scapular attachment. He remained in this condition for two days, when he was found and brought to the city. I was sent for, and found one mass of offensive putrefaction. Above the solution of continuity, the surrounding skin was diffusively inflamed and heated. This was in the afternoon. I told his friends that he had but one chance for life, and, of course, the means were obvious, but I would defer the operation until morning, directing, in the intermediate time, the constant application of cold, evaporating lotions to the inflamed surfaces, and regulating the necessary reaction of his system. Next day at noon I proceeded to the operation. The undestroyed portion of the limb being too short to allow the use of a tourniquet, the pressure of which, moreover, could not have been well borne, I arrested the circulation by the application of a key above the clavicle, confided to the care of Dr. Prior. The arm was removed by the circular operation, and an anodyne immediately administered.

On the third day I opened the bandages, when a hot and offensive fetor was emitted. The adhesive straps had fallen off, owing to the copious flow of a thin, ichorus matter, and the edges of the wound gaped; great heat and erysipelatous inflammation existed around and above the stump. Applied wet cups to the shoulder, and a flaxseed cataplasm to the stump. Ordered nitro-saline powders every four hours, with antiphlogistic diet.

By a continuance of this course for three days, the inflammation was subdued, and healthy pus established, with a granulating process. The wound gradually healed, the ligatures coming away in due time, leaving a very excellent stump.

CASE IV.—*Incised Wound of Abdomen entering the Intestine—Escape of Fluids through the Wound—Union and Recovery.*—Stepney, aged forty-eight, received a wound from a large clasp-knife, in the abdomen, on the left side above the umbilicus, entering a fold of the jejunum. This having occurred some distance from the city, he was brought down in a boat. I found it necessary to cleanse and return the protruded bowels, and secure the edges of the gut to the external opening. He had bled profusely. Pulse feeble and quick; skin cold and clammy; slight hiccough; senses perfect. After much trouble, full reaction was restored.

*Treatment.*—Simple enema to unload the lower bowels, and to ascertain if there was internal hemorrhage. Bowels relieved; no hemorrhage. A portion of whatever was administered by the mouth, either of food or medicine, passed through the wound. He was confined to a strict antiphlogistic regimen, with occasional enemata. On the second day, high, constitutional irritation, fever, pain, and much thirst. There was ℥xvi diaphoretics and saline mixture. These subdued the urgent symptoms. The wound progressed favorably in its union, which was evident by the diminished escape of ingesta until it cicatrized perfectly.

CASE V.—*Muscular Contusion with Disruption of Fibres.*—Leurendin, a mulatto creole, aged 17, attempted, on the morning of Sept. 13th, 1843, to raise a dead hog above his head, in order to hang it upon a hook. Being unequal to the task, he lost his balance and fell backward, the hog falling over his shoulders. The strain was very severe, and he complained for several days, when a circumscribed swelling, accompanied with much pain and heat, appeared over the right scapular region. It continued to increase in dimensions and severity, when I was consulted. It had now acquired the size of a goose's egg. I applied liberally on and around the tumor, tincture iodine ℈ij to ℥i alcohol. This partially relieved the pain and tension, but did not reduce the swelling. Its application was continued two days longer, and temporarily circumscribed the walls of the cavity; but I perceived a deep-seated fluctuation, which, from day to day, approached the surface, and involved the subcutaneous texture over the entire scapula. I opened it freely by a valvular incision at its most dependent part, and by placing the patient in a favorable posture for the escape of matter and gradual pressure, it discharged about fourteen ounces of pus. In order to prevent its further diffusion through the surrounding tissue, I applied a bandage with tolerable pressure from the lower part of the thorax upward, so as to urge the matter toward the opening. By these means, in the course of a week, I emptied the sac, and then finding it indisposed to assume internally an adhesive action, I threw in, twice daily, a solution of nitrate of silver, four grains to the ounce, previously cleansing it out with castile soap and water. In a fortnight it was entirely healed.

CASE VI.—*Gangrene Cured by the Use of Pyroligneous Acid.*—Jack, a colored man, was sent to me, August, 1824, with a request that I would amputate the hand. The condition of the patient was a sphacelation of the entire right hand, from the tip of the fingers to its carpal junction. The swelling was enormous. This state was the result of a phlegmonous abscess, at first neglected, and ultimately maltreated by the overseer, who, too frequently, from motives of "economy," is installed surgeon and physician to the estate. Things bore an unpromising aspect, and very little else was to be hoped for than a resort to amputation, not only on account of the destruction already present, but from the menacing prognosis; besides, the thermometer at 90° Fahr., offered no encouragement. I determined, however, to save, if I could, and operate, if I must,—a rule which I always adopt. I commenced with the ordinary means of combating such sequence to inflammation, and tried the carrot, yeast, and bark poultices, with nitric acid washes, and other remedies. These of course mitigated suffering, and, to some extent, improved appearances, yet nothing definite was accomplished

*The pyroligneous acid* was attracting attention as an antiseptic, and I had frequently used it for domestic purposes as a preservative for meats, during the summer, even when the article was what epicures call a "little short." It suggested itself to my mind that it could be equally serviceable remedially. A solution one-fourth acid, and three-fourths water, was prepared, and, removing all other applications, I kept the hand enveloped constantly in cloths saturated with the wash. In less than twenty-four hours the fetor, hitherto insupportable, was removed, and the face of the wound improved; sloughing commenced from a well-defined line of demarcation, and, by continuing the use of this prescription, in due time sloughing was completed, granulation and healthy pus substituted, and, finally, cicatrization perfected a cure. The hand was, of course, not so symmetrical, but by no means deformed, and certainly useful, as well as more ornamental than a stump. I have since used the remedy with equal satisfaction. There is a great choice in the article.

